# Leaf elemental composition of *Cleome gynandra* L. as influenced by giant kelp extract and kraal manure application

**DOI:** 10.3389/fpls.2026.1773934

**Published:** 2026-03-05

**Authors:** Naledi Makhubalo, Chuene Victor Mashamaite, Alen Manyevere

**Affiliations:** 1 Department of Agronomy, University of Fort Hare, Alice, South Africa; 2 Agricultural Research Council, Vegetables, Industrial and Medicinal Plants (ARC-VIMP), Tshwane, South Africa

**Keywords:** biostimulants, indigenous leafy vegetables nutrients, nutrient accumulation, organic manure, spider plant

## Abstract

*Cleome gynandra* L. (spider plant) is a traditional leafy vegetable that contains minerals, vitamins, proteins, and phytochemical compounds. However, little is known about the effects of sole KM (kraal manure), GKE (giant kelp extract), and GKE + KM on spider plant leaf mineral composition. This study aimed to determine the effect of sole GKE, KM, and GKE + KM on leaf mineral composition. It is hypothesized that 1 mL/L GKE supplemented with 30 kg/345 m^2^ KM will influence leaf nutrient composition. This hypothesis was tested using a split-plot design across two growing seasons at the University of Fort Hare research farm, to assess treatment-specific effects on macro- and micronutrient accumulation in spider plant leaves. The main plots comprised three levels of KM (0, 30, and 60 kg/345 m^2^) and subplots comprised five levels of GKE (0, 1, 2, 3, and 4 mL/L), each replicated three times. A two-way analysis of variance test was performed using JMP Pro 18 statistical software package to determine the effects of various levels of GKE and KM application on leaf chemical parameters. Canonical correspondence analysis was performed using R Studio version 4.1.2 (2024). Results showed that application of 30 kg/345 m^2^ KM in season 1 increased (p < 0.05) nitrogen, calcium, magnesium, potassium, sodium, iron, manganese, and zinc. Spider plant treated with 2 mL/L GKE showed increased levels of iron, manganese, and zinc in both summer seasons. Additionally, the combination of 2 mL/L GKE + 30 kg/345 m^2^ KM increased leaf nitrogen, iron, manganese, and zinc. This study confirms that GKE, KM, and GKE + KM play a significant role in leaf mineral uptake.

## 1 Introduction

*Cleome gynandra* L. (hereafter, spider plant) is an underutilized plant used for various purposes, including human food and medicine, as well as animal feed (Omondi et al., 2017; Vatsalya et al., 2025). Its leaves are rich in various essential macro and micronutrients such as calcium (2209.8 mg.100g^-1^), iron (35.7 mg.100g^-1^), zinc (8.4 mg.100g^-1^), potassium (410 mg. 100g^-1^), magnesium (86 mg.100g^-1^), sodium (34 mg.100g^-1^), phosphorus (12 mg.100g^-1^), and manganese (10−37.5 mg.100g^-1^). Additionally, it also includes vitamins A, B, and C (6.7–18.9, 0.08–0.1, and 127–484 mg.100g^-1^) and protein (2.6–6.0 mg.100g^-1^) (Chweya, 1990; Van Den Heever and Venter, 2007; Van Der Walt et al., 2009; Mishra et al., 2011; Schönfeldt and Pretorius, 2011; Jinazali et al., 2017; Mashamaite et al., 2022a; Moyo and Aremu, 2022). Due to its rich nutritional and therapeutic properties (Mashamaite et al., 2022), spider plant has the potential to contribute significantly to food security, improved nutrition, and overall well-being (Moyo and Aremu, 2022). Nutrient-dense crops like spider plant can help address several facets of malnutrition simultaneously, making them essential in combating hidden hunger in Southern Africa (Mgwenya et al., 2025). South African Modi and Mabhaudhi (2013) reported that integrating these crops into school food programs improved the nutritional condition of children by 20% within a year, compared to meals mostly consisting of imported staples. This could support the achievement of the first three 2030 Sustainable Development Goals (SDGs) Agenda: (1) No Poverty, (2) Zero Hunger, and (3) Good Health and Well-being (United Nations, 2024).

Even though it possesses exceptional multifunctional advantages, including nutritional and therapeutic properties crucial for maintaining good health and protection against illnesses, spider plant is still underutilized (Mashamaite et al., 2022). This is because most orphan crops, including spider plant, produce low economic yields and poor quality compared to main crops, which have been attributed to limited investments in research on their agronomic potential (Mabhaudhi et al., 2019; Blalogoe et al., 2020). Furthermore, because spider plants commonly grow in the wild, they are often regarded by many as agricultural weeds (Drimie and Pereira, 2016). The nutritional composition of spider plant varies considerably and is greatly affected by soil fertility, nutrient management strategies, and agroecological conditions. Understanding how eco-friendly nutrient inputs affect the elemental composition of spider plant is therefore critical for improving both yield and nutritional value (Omondi et al., 2017; Mashamaite et al., 2022). Variability in nutrient accumulation has been reported across locations and management systems, highlighting the importance of agronomic interventions to improve both yield and the nutritional value of indigenous leafy vegetables (Moyo and Aremu, 2022; Mgwenya et al., 2025). Understanding how sustainable nutrient inputs influence the elemental composition of spider plant is therefore critical for enhancing its contribution to food and nutrition security. To enhance the potential of this leafy vegetable as a future economic crop, it is essential to generate scientifically validated knowledge on improving leaf yield, productivity, and nutritional quality using eco-friendly inputs such as seaweed extracts (Petrick, 2020).

Organic nutrient inputs, such as animal manures and seaweed-based biostimulants, have received increasing attention for their role in enhancing soil fertility, nutrient availability, and plant nutrient uptake while reducing dependence on synthetic fertilizers (Eghball et al., 2002; Dordas, 2008; Godlewska et al., 2016). Kraal manure serves as a source of slow-release macro- and micronutrients and organic matter, improving soil structure and microbial activity (Miles and Manson, 1998; Matsi, 2011). Seaweed extracts, including giant kelp extract, contain essential nutrients and bioactive compounds such as phytohormones that stimulate physiological processes involved in nutrient absorption and assimilation (Godlewska et al., 2017; Sosnowski et al., 2023). Although these inputs have been investigated in several crops, their combined influence on the nutritional quality of indigenous leafy vegetables remains poorly documented.

The open-field experiments conducted in the current study were the same as those in the study by Makhubalo (2025), which examined the impact of giant kelp extract (GKE) combined with kraal manure (KM) on the growth, yield, and biomass of spider plants. Comprehensive details on the study design and treatments are discussed in the methodology section. Briefly, the study findings showed that different levels of GKE and KM significantly affected plant growth parameters, including stem diameter, chlorophyll content, leaf number, root biomass, and shoot biomass of this leafy vegetable. Giant kelp extract is an abundant source of phosphorus, iron, potassium, sodium, zinc, copper, calcium, manganese, vitamins C and E, and tannins (Godlewska et al., 2016; Godlewska et al., 2017; Kristanto et al., 2021). Therefore, incorporating both GKE and KM may stimulate certain physiological processes, thereby improving crop development and growth, and reducing the need for chemical fertilizers without negatively affecting agricultural productivity.

Although the application of GKE and KM improved spider plant growth and productivity (Makoro, 2015; Makhubalo, 2025), limited information is available on how these inputs, applied individually or in combination, affect the leaf elemental composition of spider plant under open-field conditions. Moreover, most existing studies focus on growth responses, with comparatively fewer reports examining nutrient accumulation and its relationship with vegetative performance across seasons. While the study by Makhubalo (2025) focused on assessing the effects of GKE and KM on the growth, yield, and biomass of spider plant, this paper builds on the same trial by examining how sole GKE, KM, and GKE + KM influence the crop’s elemental composition. This continuity in experimental design allows for a comprehensive evaluation of both agronomic performance and nutritional quality. Improving the concentration of key micronutrients such as iron, zinc, and manganese in spider plant leaves through sustainable agronomic practices may help address micronutrient deficiencies prevalent in resource-limited communities (Schönfeldt and Pretorius, 2011; Nandal and Solanki, 2021; Mgwenya et al., 2025). Agronomic biofortification using eco-friendly inputs, therefore, represents a promising strategy for enhancing the nutritional value of indigenous leafy vegetables without compromising environmentalz sustainability. Therefore, the objective of this study was to determine whether applying GKE and KM would affect the nutrient content of spider plants. It is hypothesized that the application of GKE and KM, individually or in combination, will significantly affect the elemental composition of spider plant leaves.

## 2 Methodology

### 2.1 Description of study site

Two field trials were conducted at the University of Fort Hare Research Farm in the Eastern Cape Province, South Africa. The farm is situated at a latitude of 32.79° S, a longitude of 26.84° E, and at about 522 m above sea level (Moeletsi et al., 2022). The first trial was conducted during the summer season from January – March 2024 (season 1), while the second season was conducted the following year, 2025 (season 2). According to Moeletsi et al. (2022), the area receives 54.10 – 15.75 mm of rainfall during the summer and autumn (**Table 1**). The soil was identified as Calcic Cambisol (Soil Classification Working Group, 1991). The chemical composition of the soil utilized before beginning the experiments was as described (**Table 2**).

**Table 1:** Total rainfall and average temperature in Alice during the year 2024.

	Mean temperature °C		Total rainfall (mm)
Month	Daily minimum temperature	Daily maximum temperature	
January	15.81	30.39	46.99
February	14.27	31.58	21.84
March	15.04	31.89	54.10
April	12.28	26.10	51.56
May	9.37	27.92	15.75

**Table 2:** Initial soil chemical properties composition for seasons 1 and 2.

Soil chemical properties	Season 1	Season 2
pH (KCl)	5.90	5.70
Boron (mg/kg)	0.35	0.36
Carbon (%)	1.09	0.80
Calcium cmol (+)/kg	6.21	5.65
Copper (mg/kg)	0.61	0.57
Iron (mg/kg)	8.35	6.50
Potassium (mg/kg)	264.00	125.00
Magnesium cmol (+)/kg	2.58	2.58
Manganese (mg/kg)	8.84	8.46
Sodium (mg/kg)	39.00	48.00
Phosphorus (citric acid) (mg/kg)	77.00	50.00
Zinc (mg/kg)	0.47	0.40

### 2.2 Experimental research design and treatments

The study was carried out using a split-plot experimental design with main plots containing three KM levels (0, 30, and 60 kg/345 m^2^), each applied once. The subplots comprise five concentrations of GKE (0, 1, 2, 3, and 4 mL/L) combined with 1 L of deionized water, each replicated three times. A liquid GKE was acquired from Kelp Blue Namibia (Pty) Ltd, while cow manure was randomly sourced from communities in Ntselamanzi village, near the University of Fort Hare. Chemical analysis results for both KM and GKE are shown in **Tables 3**–**5**. The overall size of the field was 15 m x 23 m, with each main plot measuring 15 m x 7 m, being divided by a 1 m gap. The dimensions of each subplot (net plot) were 3 m x 2 m, with a separation of 0.5 m between them. The spider plant seeds were sown with an inter-row spacing of 0.2 m and an intra-row spacing of 0.3 m at a depth of 0 − 5 cm. The field had 45 experimental units with 15 treatments, each containing 40 plants, resulting in a total of 1800 plants per hectare. The KM levels were administered once before planting, whereas the GKE levels were applied immediately post-planting every 14 days, for in a total of six applications over the growth period. The field was rainfed in terms of water requirements.

**Table 3:** Giant kelp extract mineral composition.

Component	Value (mg/L)
Boron	3.67
Calcium	137
Copper	0.23
Iron	0.35
Potassium	3464
Magnesium	244
Manganese	0.15
Sodium	1268
Phosphorus	405
Zinc	0.96

**Table 4:** Giant kelp extract phytohormone composition Ali et al. (2018).

Component	Value (μg/g FW)
Gibberellins	0.65
Cytokinins	0.63
Indole acetic acid	0.72
Abscisic acid	0.13
Salicylic acid	1.87
Trans-Jasmonic acid	0.20

**Table 5:** Kraal manure chemical properties composition.

Component	Value
pH	7.71
Organic Matter (%)	9.11
Organic Carbon (%)	5.30
Nitrogen (g/kg)	13.60
Phosphorus (%)	0.43
Potassium (%)	0.78
Calcium (%)	0.96
Magnesium (%)	0.28
Sodium (mg/kg)	1228.00
Iron (mg/kg)	1199.57
Copper (mg/kg)	6.50
Zinc (mg/kg)	93.29
Manganese (mg/kg)	169.95
Boron (mg/kg)	13.13

### 2.3 Chemical composition analyses

After eight weeks of planting, freshly harvested spider plant leaves from both seasons were collected from each treatment (5 plants per treatment) and placed in a brown bag and oven-dried at 60 °C for 72 hours (Sowunmi and Oyedeji, 2019). Additionally, leaf samples were subsequently milled using a Wiley mill, passed through a 1 mm sieve, and stored in plastic bags before laboratory examination (Matizha et al., 2001; Mpongwana et al., 2023). Leaf nutrient analyses were conducted following standard laboratory protocols, with instrument calibration and quality control performed prior to sample analysis, as described below. Leaf samples were digested with hydrogen peroxide, sulfuric acid, selenium powder mixture (4:1 v/v), and salicylic acid to determine micro- and macro-elements. Nitrogen and phosphorus were determined by the Kjeldahl technique and the molybdenum blue method using an Ultraviolet visible -Vis spectrophotometer (Sherwood 420) at an absorbance of 650 nm and 880 nm, respectively. While potassium, calcium, magnesium, manganese, iron, zinc, and sodium in plant samples were measured using the atomic absorption spectrometry (iCE™ 3500) at a wavelength of 766.5, 422.7, 285.2, 248.3, 248.3, 213.9, 589.0 nm as reported by Okalebo et al. (2002).

### 2.4 Statistical data analyses

A two-way analysis of variance (2-way ANOVA) test was performed using JMP Pro 18 statistical software package to determine the effects of various levels of GKE and KM application on selected leaf chemical parameters (i.e., nitrogen, phosphorus, potassium, calcium, etc.). Means were separated using the Tukey HSD test at a 5% probability level. Multivariate relationships between leaf nutrient composition and growth parameters were explored using canonical correspondence analysis (CCA). These analyses were done using the “vegan” and “labdsv” packages. This approach was chosen to evaluate treatment-driven patterns in nutrient uptake and to identify associations between mineral elements and vegetative growth variables. Multivariate graphs were generated using R with the “ggplot2” package. All multivariate analyses were conducted in RStudio version 4.1.2 (2024). Assumptions of normality and homogeneity of variance were assessed prior to analysis.

## 3 Results

### 3.1 Effect of KM and GKE on leaf nitrogen content from spider plant (seasons 1 and 2)

**Figure 1:**
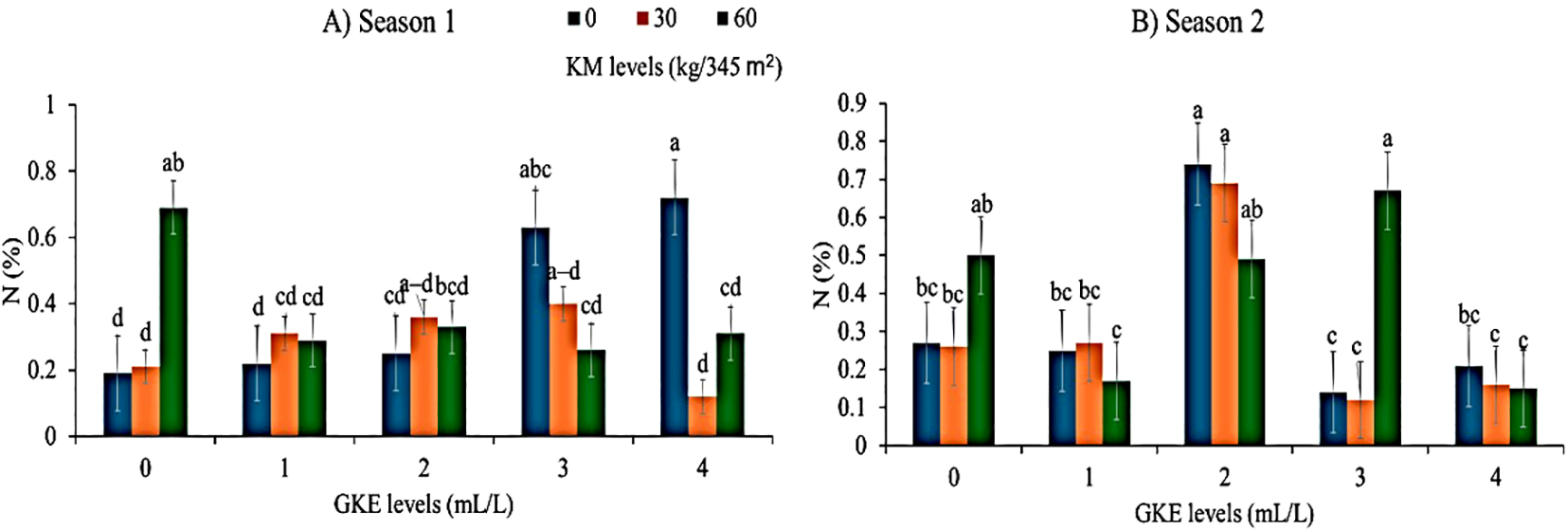
The effects of giant kelp extract (GKE) and kraal manure (KM) on nitrogen (N) content of spider plant leaves in **(A)** season 1, and **(B)** season 2. Different letters indicate significant differences between treatments at p = 0.05 (ANOVA and Tukey’s HSD).

**Figure 2:**
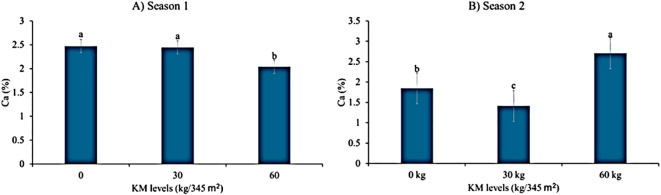
The effects of giant kelp extract (GKE) and kraal manure (KM) on calcium (Ca) of spider plant leaves in both **(A)** season 1 and **(B)** season 2. Different letters indicate significant differences between treatments at p = 0.05 (ANOVA and Tukey’s HSD).

Leaf nitrogen showed a significant difference (p <no><</no> 0.05) when spider plant was applied with KM in both seasons. A noticeable increase in leaf nitrogen was found in both seasons when spider plant was treated with 60 kg/345 m^2^ KM (**Figures 1A, B**). The addition of GKE in season 1 did not affect nitrogen content (p > 0.05) (**Table 6**). **Figure 1A** indicated that, during season 1, nitrogen concentration remained stable when 1 mL/L and 2 mL/L GKE were applied individually. Similarly, in season 2, the application of 1, 2, and 3 mL/L GKE alone did not affect nitrogen (**Figure 1B**). The administration of the treatment combination of 3 mL/L GKE + 60 kg/345 m^2^ KM resulted in an increased leaf nitrogen (**Figure 1A**). Leaf nitrogen significantly differed (p <no><</no> 0.001) when spider plant was treated with GKE in season 2 (**Table 6**). As the application of GKE levels increased from 1 mL/L to 2 mL/L, leaf nitrogen increased (**Figure 1B**). Furthermore, when spider plant was treated with the GKE + KM combination, nitrogen was highly significant (p <no><</no> 0.001) in both seasons (**Table 7**). Spider plant treated with 3 mL/L GKE + 60 kg/345 m^2^ KM showed an increased leaf nitrogen in season 2 (**Figure 2B**)

**Table 6:** Effect of giant kelp extract (GKE) supplemented with kraal manure (KM) on spider plant growth and yield parameters during summer seasons 2024 and 2025.

Summer season 2024 (Season 1)										Summer season 2025 (Season 2)								
Treatments	N	Ca	Mg	K	Na	Iron	Mn	Zn	P	N	Ca	Mg	K	Na	Iron	Mn	Zn	P
KM levels (kg/345 m^2^)																		
0	0.40^a^	2.47^a^	0.48^ab^	0.39^b^	393.39^b^	511.29^a^	67.12^a^	53.81^b^	1.04^a^	0.32^ab^	1.85^b^	0.43^a^	1.11^a^	623.23^a^	624.23^a^	195.02^a^	162.75^b^	1.08^a^
30	0.28^b^	2.45^a^	0.51^a^	1.06^a^	493.83^a^	317.31^b^	40.27^b^	41.34^b^	1.06^a^	0.30^b^	1.41^c^	0.46^a^	1.05^a^	502.38^b^	660.53^a^	135.72^ab^	204.10^a^	1.08^a^
60	0.38^ab^	2.04^b^	0.42^b^	0.95^a^	444.93^ab^	308.63^b^	35.56^b^	192.67^a^	1.05^a^	0.40^a^	2.71^a^	0.43^a^	0.69^b^	563.51^ab^	629.53^a^	58.30^b^	169.01^b^	1.10^a^
P value	^*^	^***^	**	^***^	^*^	***	^***^	***	ns	*	***	ns	***	**	ns	**	***	ns
GKE levels (mL/L)																		
0	0.36^a^	2.44^a^	0.49^a^	0.88^a^	451.14^a^	434.73^b^	48.33^b^	150.25^a^	1.05^a^	0.35^b^	1.99^a^	0.47^a^	0.87^a^	685.25^a^	570.72^bc^	129.56^ab^	136.48^b^	1.11^a^
1	0.27^a^	2.28^a^	0.41^a^	0.72^a^	471.83^a^	275.48^c^	41.35^b^	49.63^b^	1.04^a^	0.23^bc^	2.18^a^	0.48^a^	0.93^a^	624.12^ab^	698.25^ab^	48.19^b^	247.93^a^	0.99^a^
2	0.32^a^	2.41^a^	0.49^a^	0.93^a^	434.74^a^	596.75^a^	71.94^a^	47.70^b^	1.02^a^	0.64^a^	1.96^a^	0.46^a^	0.91^a^	512.56^bc^	523.60^c^	53.11^b^	249.02^a^	1.11^a^
3	0.42^a^	2.28^a^	0.50^a^	0.79^a^	419.90^a^	247.00^c^	41.99^b^	143.88^a^	0.95^a^	0.31^bc^	1.95^a^	0.40^a^	0.96^a^	459.54^c^	795.46^a^	216.80^a^	130.08^b^	1.12^a^
4 L	0.38^a^	2.18^a^	0.47^a^	0.70^a^	409.31^a^	341.41^bc^	34.63^b^	88.24^b^	1.18^a^	0.18^c^	1.86^a^	0.37^a^	1.10^a^	533.74^bc^	601.70^bc^	200.74^ab^	129.58^b^	1.10^a^
P value	ns	ns	ns	ns	ns	***	***	***	ns	***	ns	ns	ns	***	**	**	**	ns
CV (%)	34.29	12.50	17.02	27.50	17.12	27.34	30.37	39.36	23.81	29.41	12.56	20.45	24.21	17.90	19.40	90.65	6.87	24.77

Means in a column with different letters are significantly different at P < 0.05*, P < 0.01**, and P < 0.001***. ns, not significant. GKE, Giant kelp extract; KM, Kraal manure.

**Table 7:** Interaction effects of giant kelp extract and kraal manure (GKE + KM) on spider plant leaf nutrients during 2024 and 2025 summer seasons.

Summer 2024 (Season 1)																																										
N (%) Treatments						Ca (%)					Mg (%)					K (%)				Na (mg/kg)				Fe (mg/kg)					Mn (mg/kg)					Zn (mg/kg)				P (%)				
KM levels (kg/345 m^2^)																																										
GKE Levels (mL/L)		0	30		60	0	30		60	0		30		60	0		30		60		0	30	60		0		30	60		0		30	60	0		30	60		0		30	60
0		0.19^d^	0.21^d^		0.69^ab^	2.79^a^	2.50^ab^		2.03^ab^	0.57^a^		0.53^ab^		0.37^ab^	0.16^d^		1.47^a^		1.01^ab^		434.77^a^	422.40^a^	496.45^a^		640.94^ab^		373.36^bcd^	289.88^d^		44.07^b^		66.42^b^	34.49^b^	71.82^bc^		46.67^c^	332.25^a^		1.15a		1.00^a^	1.01^a^
1		0.22^d^	0.31^cd^		0.29^cd^	2.62^a^	2.32^ab^		1.91^ab^	0.47^ab^		0.46^ab^		0.31^b^	0.27^cd^		1.06^ab^		0.82^abcd^		409.86^a^	542.55^a^	463.09^a^		390.65^bcd^		204.17^d^	231.62^d^		46.62^b^		39.10^b^	38.34^b^	45.93^c^		50.16^c^	52.82^c^		1.11^a^		0.92^a^	1.10^a^
2		0.25^cd^	0.36^abcd^		0.33^bcd^	2.22^ab^	2.57^a^		2.46^ab^	0.42^ab^		0.59^a^		0.46^ab^	1.06^ab^		0.73^bcd^		0.99^ab^		365.00^a^	488.06^a^	451.15^a^		903.75^a^		603.82^abc^	282.68^d^		159.48^a^		25.03^b^	31.31^b^	48.90^c^		36.94^c^	57.27^c^		1.12^a^		1.08^a^	0.88^a^
3		0.63^abc^	0.40^abcd^		0.26^cd^	2.29^ab^	2.41^ab^		2.13^ab^	0.48^ab^		0.50^ab^		0.51^ab^	0.30^cd^		1.15^ab^		0.92^abc^		360.79^a^	427.26^a^	471.65^a^		211.81^d^		214.71^d^	314.49^cd^		39.87^b^		43.39^b^	42.70^b^	54.32^c^		39.55^c^	337.76^a^		0.88^a^		1.05^a^	0.91^a^
4		0.72^a^	0.12^d^		0.31^cd^	2.44^ab^	2.43^ab^		1.67^b^	0.48^ab^		0.48^ab^		0.46^ab^	0.16^d^		0.91^abc^		1.02^ab^		396.51^a^	488.90^a^	342.52^a^		409.28^bcd^		190.50^d^	424.45^bcd^		45.53^b^		27.42^b^	30.94^b^	48.10^c^		33.40^c^	183.23^b^		0.993^a^		1.26^a^	1.37^a^
P value				***				ns					ns					***				ns				***					***				***					ns		
CV (%)				34.29				12.50					17.02					27.50				17.12				27.34					30.37				39.36					23.81		
Summer 2025 (Season 2)																																										
0	0.27^bc^		0.26^bc^		0.50^ab^	1.87^c^	1.38^cd^		2.71^a^	0.44^a^		0.45^a^		0.53^a^	1.04^ab^		0.88^ab^		0.70^b^		1038.00^a^	325.50^c^	629.20^b^		426.50^cd^		566.70^bcd^	718.90^bcd^		272.81^ab^		44.57^b^	71.31^b^	331.28^b^		46.42^cd^	31.74^cd^		1.05^a^		1.17^a^	1.11^a^
1	0.25^bc^		0.27^bc^		0.17^c^	1.90^bc^	1.86^c^		2.78^a^	0.47^a^		0.50^a^		0.48^a^	1.15^ab^		1.03^ab^		0.60^b^		591.30^bc^	718.00^b^	563.10^bc^		739.70^bc^		640.30^bcd^	714.80^bcd^		48.05^b^		35.99^b^	60.54^b^	54.85^c^		314.09^b^	374.84^a^		1.10^a^		0.94^a^	0.94^a^
2	0.73^a^		0.69^a^		0.49^ab^	1.89^bc^	1.29^cd^		2.70^a^	0.44^a^		0.48^a^		0.45^a^	0.98^ab^		0.84^ab^		0.90^ab^		429.30^bc^	526.40^bc^	582.00^bc^		379.20^cd^		570.80^bcd^	620.80^bcd^		56.72^b^		34.22^b^	68.38^b^	43.71^cd^		313.93^b^	389.43^a^		1.05^a^		1.15^a^	1.15^a^
3	0.14^c^		0.12^c^		0.67^a^	1.68^cd^	1.48^cd^		2.70^a^	0.40a		0.45^a^		0.36^a^	1.25^ab^		1.01^ab^		0.62^b^		453.60^bc^	573.00^bc^	352.00^c^		1228.30^a^		651.90^bcd^	506.20^bcd^		523.94^a^		76.64^b^	49.83^b^	53.20^c^		302.03^b^	35.01^cd^		1.05^a^		1.20^a^	1.11^a^
4	0.21^bc^		0.16c		0.15^c^	1.89^bc^	1.05^d^		2.65^ab^	0.41^a^		0.39^a^		0.31^a^	1.14^ab^		1.52^a^		0.63^b^		604.00^bc^	369.00^c^	628.20^bc^		347.50^d^		873.00^ab^	584.70^bcd^		73.58^b^		487.19^a^	41.46^b^	330.69^b^		44.02^cd^	14.03^d^		1.17^a^		0.93^a^	1.21^a^
P value				***				ns					ns					***				***				^***^					***				***					ns		
CV (%)				29.41				12.56					20.45					24.21				17.90				19.40					90.65				6.87					24.77		

Means in a column with different letters are significantly different at P < 0.05*, P < 0.01**, and P < 0.001***. ns, not significant. GKE, Giant kelp extract; KM, Kraal manure.

### 3.2 Response of leaf calcium during seasons 1 and 2 to different GKE and KM levels

As shown by **Table 6**, leaf calcium showed a highly significant difference (p < 0.001) when KM levels were applied to spider plant for both seasons. **Figure 2A** showed that the control had a constant calcium level with spider plant treated with 30 kg/345 m^2^ KM, but as KM levels increased, calcium decreased in season 1. n season 2, applying 30 kg/345 m² KM resulted in lower calcium levels than the control; however, calcium levels increased as KM application rates increased (**Figure 2B**). Spider plant treated with GKE showed no significant differences (p > 0.05) in calcium concentration for both seasons (**Table 6**). Results further showed no significant difference (p > 0.05) in leaf calcium when sole GKE and GKE + KM were administered to spider plant (**Tables 6**, **7**).

### 3.3 Response of leaf magnesium to different GKE and KM levels during seasons 1 and 2

**Figure 3:**
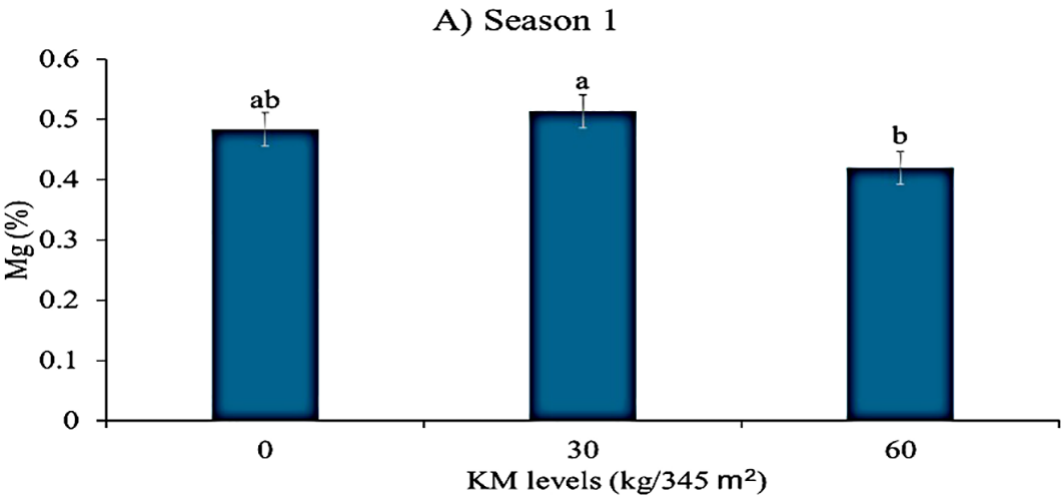
The effects of giant kelp extract (GKE) and kraal manure (KM) on magnesium (Mg) content in spider plant leaves in (A) season 1. Different letters indicate significant differences between treatments at p = 0.05 (ANOVA and Tukey’s HSD).

**Table 6** shows that applying KM to spider plants produced a highly significant (p < 0.01) effect on leaf magnesium content during season 1. As illustrated in **Figure 3**, plants treated with 30 kg/345 m² KM alone had higher leaf magnesium levels than those treated with 60 kg/345 m² KM. In contrast, KM application had no significant effect (p > 0.05) on leaf magnesium content in season 2 (**Table 6**). Similarly, the sole application of GKE and GKE + KM to spider plant did not significantly affect leaf magnesium in both seasons (**Tables 6**, **7**).

### 3.4 Response of leaf potassium content to different GKE and KM during seasons 1 and 2

**Figure 4:**
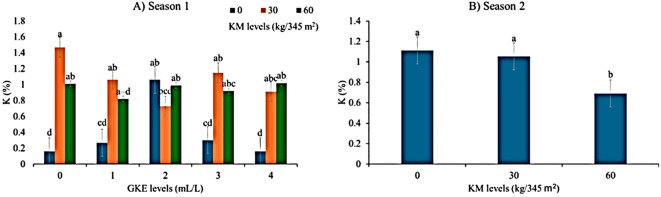
The effects of giant kelp extract (GKE) and kraal manure (KM) on potassium (K) of spider plant leaves in **(A)** season 1 and **(B)** season 2. Different letters indicate significant differences between treatments at p = 0.05 (ANOVA and Tukey’s HSD).

Findings showed the application of KM in both seasons on spider plant resulted in a highly significant difference (p < 0.001) in leaf potassium (**Table 6**). Thus, the application of 30 kg/345 m^2^ KM to spider plant during season 1 increased leaf potassium compared to the control, but leaf potassium remained the same as the KM amounts increased to 60 kg/345 m^2^ KM (**Figure 4A**). No significant difference (p > 0.05) was observed in leaf potassium in both seasons when GKE was applied to the plant (**Table 6**). Leaf potassium remained constant when spider plant was treated with 1 mL/L, 3 mL/L GKE, and 4 mL/L GKE alone (**Figure 4A**). Season 1 showed a highly significant difference (p < 0.001) in response to GKE + KM. Spider plant treated with 1 mL/L GKE + 30 kg/345 m^2^ KM had a higher leaf potassium concentration than those treated with 1 mL/L GKE + 60 kg/345 m^2^ KM (**Figure 4A**). Season 2 showed that spider plant treated with GKE + KM had no significant difference (p > 0.05) (**Table 7**).

### 3.5 Response of KM and GKE on sodium content of spider plant (seasons 1 and 2)

**Figure 5:**
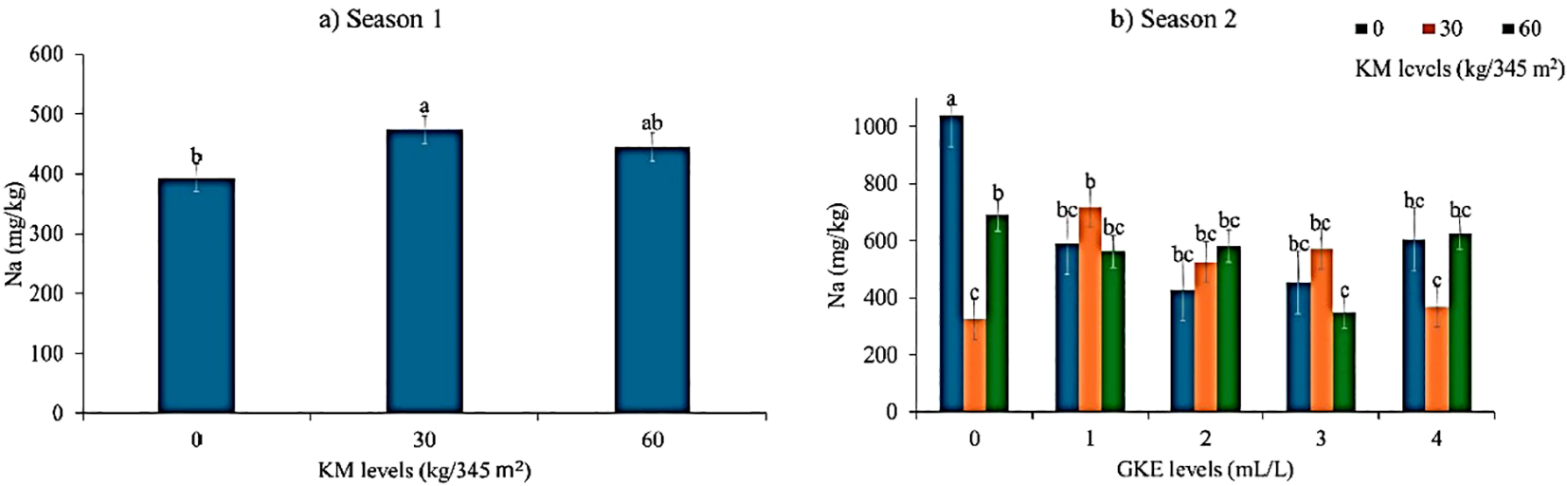
The effects of giant kelp extract (GKE) and kraal manure (KM) on sodium (Na) of spider plant leaves in **(a)** season 1 and **(b)** season 2. Different letters indicate significant differences between treatments at p = 0.05 (ANOVA and Tukey’s HSD).

A statistical difference (p < 0.05) in leaf sodium was found in both seasons when KM was applied to spider plant. This was noticeable as KM levels increased in both seasons; leaf sodium levels increased (**Figure 5**). No significant difference (p > 0.05) in leaf sodium was found when spider plant was applied with GKE in season 1 (**Table 6**). A significant variation (p > 0.05) in leaf sodium content was observed when spider plant was treated with GKE during season 2 (**Table 6**). In contrast, no significant difference was observed when spider plant was treated with the combined GKE + KM treatment in season 1 (**Table 6**). However, leaf sodium levels showed a statistical difference when spider plants received the combined GKE + KM treatment in season 2 (**Table 7**). This trend was illustrated in **Figures 5B**, which showed that leaf sodium levels increased when spider plants were treated with 1 mL/L GKE + 30 kg/345 m² KM, but decreased when 3 mL/L GKE + 60 kg/345 m² KM was applied.

### 3.6 Response of leaf iron content when spider plant was treated with KM and GKE in seasons 1 and 2

**Figure 6:**
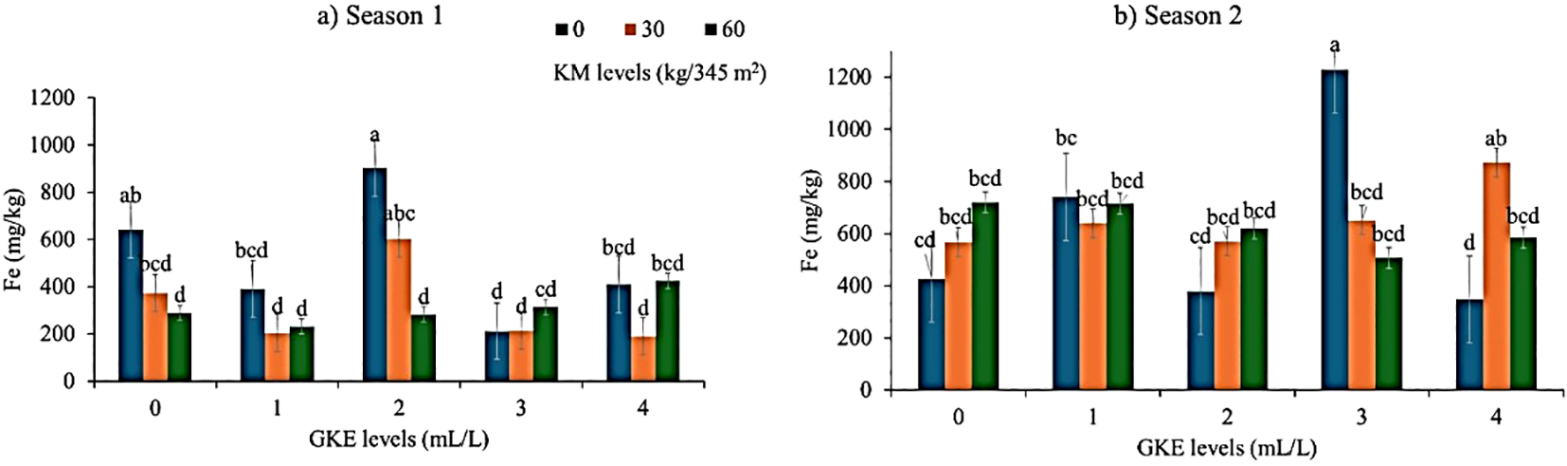
The effect of giant kelp extract (GKE) and kraal manure (KM) on iron (Fe) of spider plant leaves in **(a)** season 1, and **(b)** season 2. Different letters indicate significant differences between treatments at p = 0.05 (ANOVA and Tukey’s HSD).

A highly significant (p < 0.001) leaf iron was found when spider plant was treated with KM in season 1 (**Table 6**). Meanwhile, **Figure 6A** showed that leaf iron concentration decreased as KM application alone increased. However, season 2 did not result in any significant difference (p > 0.05) when spider plant was treated with KM (**Table 6**). Thus, as KM levels increased, iron remained constant (**Figure 6B**). Spider plant treated with GKE in both seasons showed a highly significant effect (p < 0.001) in leaf iron for both seasons (**Table 6**). Such that, as 2 mL/L GKE was administered in season 1 (**Figure 6A**), leaf iron increased, while spider plant treated with 3 mL/L GKE alone had the highest leaf iron in season 2 (**Figure 6B**). Meanwhile, spider plant treated with GKE + KM showed a highly significant difference (p < 0.001) in both seasons (**Table 7**). Season 1 (**Figure 6A**) showed that application of 2 mL/L GKE + 30 kg/345 m^2^ KM increased, but when 2 mL/L GKE + 60 kg/345 m^2^ KM was applied to spider plant, leaf iron decreased. The combination of 4 mL/L GKE + 3 kg/345 m² KM resulted in an increase in leaf iron, whereas applying 3 mL/L GKE + 60 kg/345 m² KM led to a decrease in leaf iron (**Figure 6B**).

### 3.7 Response of spider plant leaf manganese to different GKE and KM levels in seasons 1 and 2

**Figure 7:**
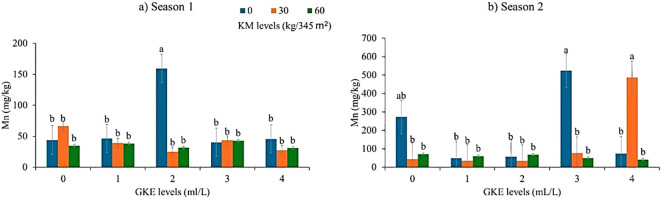
The effect of giant kelp extract (GKE) and kraal manure (KM) on manganese (Mn) of spider plant leaves in **(a)** Season 1 and **(b)** Season 2. Different letters indicate significant differences between treatments at p = 0.05 (ANOVA and Tukey’s HSD).

The results showed that leaf manganese content in spider plant leaves had a highly significant difference (p < 0.001) when KM alone, GKE alone, and GKE + KM alone were applied to spider plant (**Tables 6**, **7**). Both seasons showed that as KM levels increased, leaf manganese remained constant (**Figure 7**). Additionally, leaf manganese in season 1 increased when spider plant was applied with 2 mL/L GKE (**Figure 7A**). In season 2 spider plant revealed an increase in leaf manganese when treated with 3 mL/L GKE (**Figure 7B**). Spider plant resulted in an increased leaf manganese in season 2 when treated with 4 mL/L GKE + 30 kg/345 m^2^ KM (**Figure 7B**).

### 3.8 Response of zinc and phosphorus content during seasons 1 and 2 when GKE and KM were administered to spider plant

**Figure 8:**
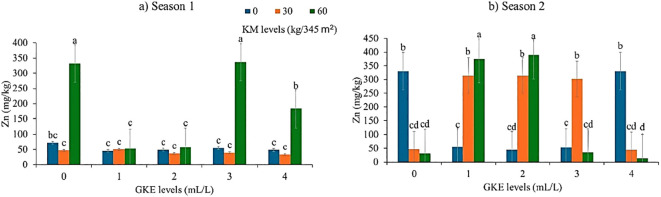
The effect of giant kelp extract (GKE) and kraal manure (KM) on zinc (Zn) of spider plant leaves in **(a)** Season 1 and **(b)** Season 2. Different letters indicate significant differences between treatments at p = 0.05 (ANOVA and Tukey’s HSD).

The findings indicated that the application of GKE alone, KM alone, or their combined treatment significantly affected zinc levels (p < 0.001), as presented in **Tables 6**, **7**. Zinc levels increased when 60 kg/345 m² KM was applied (**Figure 8A**). However, in season 2, zinc levels remained constant as KM levels increased (**Figure 8B**). Spider plant treated with 4 mL/L GKE showed an increased leaf zinc content (**Figure 8B**). Results further show that in season 1, as GKE + KM levels increased from 3 mL/L GKE + 30 kg/345 m^2^ KM alone to 3 mL/L GKE + 60 kg/345 m^2^ KM alone, zinc increased (**Figure 8A**). Similarly, an increase in zinc was observed in season 2 when GKE + KM levels increased from 30 kg/345 m^2^ KM + 1 mL/L GKE to 1 mL/L GKE + 60 kg/345 m^2^ KM (**Figure 8B**). Furthermore, there were no significant differences (p > 0.05) in leaf phosphorus when KM alone, GKE alone, and GKE + KM were applied to spider plant in both seasons (**Tables 6**, **7**).

In general, significant GKE + KM interaction effects were observed for several leaf mineral elements, indicating that the influence of GKE on nutrient accumulation depended on the level of kraal manure applied. For example, moderate KM levels (30 kg/345 m²) combined with intermediate GKE concentrations enhanced the uptake of iron, manganese, and zinc more effectively than either input applied alone.

### 3.9 Canonical correspondence analysis

**Figure 9:**
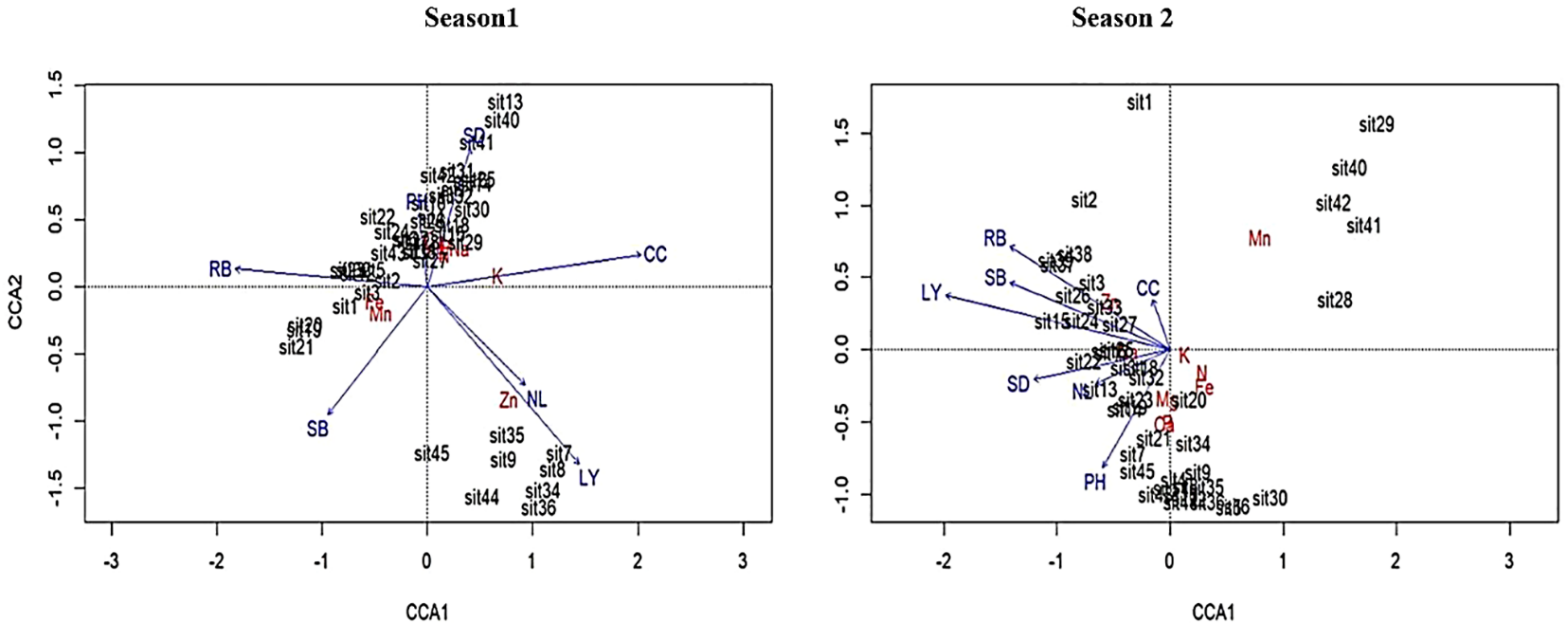
Canonical correspondence analysis (CCA) illustrating the relationship between plant growth parameters and nutrient content when spider plant was treated with KM and GKE. The blue arrow lines indicate plant growth parameters: RB (root biomass), SB (shoot biomass), CC (chlorophyll content), LY (leaf yield), NL (number of leaves), SD (stem diameter), and PH (plant height), while the red text indicate leaf nutrients: N (nitrogen), P (phosphorus), K (potassium), Zn (Zinc), Fe (Iron), Ca (calcium), Mg (magnesium), Mn (Manganese), and Na (sodium).

The CCA analysis was performed to thoroughly assess the correlation between plant growth parameters and leaf nutrient attributes at a significance threshold of p < 0.05 (**Figure 9**). The CCA 1 (season 1) showed that leaf yield and the number of leaves strongly correlated with zinc. While chlorophyll content positively correlated with potassium. Additionally, stem diameter and plant height positively correlated with sodium, phosphorus, nitrogen, and magnesium. Furthermore, CCA 2 (season 1) demonstrated that root biomass correlated positively with zinc and iron, but shoot biomass correlated negatively. Moreover, CCA 2 (season 2) showed that root biomass, shoot biomass, leaf yield, and chlorophyll content were positively correlated with zinc. However, stem diameter, number of leaves, and plant height were negatively correlated with sodium.

## 4 Discussion

The current study demonstrated a significant difference in nitrogen content when KM was applied to spider plant leaves in both seasons. For both seasons, control did not significantly differ from spider plant treated with 30 kg/345 m^2^ KM; however, as KM levels increased, leaf nitrogen increased. These results are consistent with Mahabub et al. (2016), who reported a significant variation in leaf nitrogen content of mungbean (*Vigna radiata*) following the application of 10 t KM ha^-^¹, whereas the lowest nitrogen content was recorded in the 0 t KM ha^-^¹ (control) treatment.This increase can be ascribed to the available nitrogen (13.6 g/kg) in KM. Luthuli et al. (2024) contradicted these findings, reporting that total nitrogen in tomato (*Solanum lycopersicum* L.) did not significantly differ when KM was applied. This could be because the study was conducted in an enclosed environment while the plants were treated with growing media. Thus, diverse microbial populations may be absent from glasshouse growth medium, which may affect processes such as nitrogen fixation, mineralization, and immobilization (Grunert, 2017).

Findings showed that adding GKE in season 1 did not affect leaf nitrogen content in spider plant. Sole application of 1 mL/L and 2 mL/L GKE concentration resulted in a constant leaf nitrogen concentration. Likewise, these findings correspond with those of Senthuran et al. (2019), who found no significant difference in leaf nitrogen of amaranthus (*Amaranthus polygamus* L.) when foliar spraying with 10% seaweed extract (*Kappaphycus alvarezii*). In contrast, Soleimani et al (2024) found a significant difference in leaf nitrogen when olives (*Olea europaea* L.) were treated with 2.5 g/L algora seaweed extract. Additionally, results showed that applying both 30 kg/m^2^ KM + 2 mL/L GKE highly increased leaf nitrogen in both seasons. The enhanced leaf nitrogen observed under combined KM and GKE application can be explained by complementary soil and plant physiological processes. The increase in leaf nitrogen during both seasons when the treatment combination was applied could be due to the synergistic effects of KM and GKE on soil fertility and plant growth parameters (Eghball et al., 2002; Dhiman et al., 2019). The application of 30 kg/m² KM likely enhanced soil nitrogen availability through the mineralization of nitrogen, thereby increasing plant-available nitrogen. Thus, the phytohormones present in GKE, including cytokinins and auxins, stimulated plant growth and promoted nitrogen uptake and assimilation (Sosnowski et al., 2023).

In terms of calcium, the results showed a significant difference in both seasons when KM levels were applied to spider plant. This effect was observed in season 1 whereby: applying 30 kg/345 m^2^ KM increased leaf calcium, but applying 60 kg/345 m^2^ KM decreased it; however, in season 2, as KM levels increased (60 kg/345 m^2^), calcium content increased. Similarly, findings align with those of Luthuli et al. (2024), who found a significant difference in leaf calcium of tomato when fertigating with liquid KM. The increase in season 2 may be ascribed to the available calcium (10.50 g/kg) in KM, which constitutes a substantial quantity that can facilitate calcium absorption by the spider plant. In contrast, Mmadi et al. (2019) found no significant difference in leaf calcium of potatoes (*Solanum tuberosum*) when a 7:3 KM ratio was applied. This could be attributed to different plants responding differently to KM application rates. Furthermore, spider plant treated with GKE showed no significant difference in calcium for both seasons. These results agree with those of Makoro (2015), who found no significant difference between application rates of seaweed extract (*Ecklonia maxima*) control, 1:100, and 1:500 v/v in leaf calcium content of cowpea (*Vigna unguiculata*). However, Makhaye et al. (2021) found that the application of (1:100 and 1:40) seaweed extract (Kelpak^®^) enhanced spider plant and okra (*Abelmoschus esculentus*) leaf calcium content. This was attributed to the fact that calcium in GKE may have been present in a form not readily assimilated by spider plants. Calcium alginate is an organic polymer obtained from brown seaweed, namely *Macrocystis pyrifera*. The gel-forming chemical removes calcium ions (Ca²^+^), reducing their availability for plant uptake (Rahman et al., 2024). Applying both GKE + KM showed no significant difference in leaf calcium in both seasons. This binding effect limits the availability of free Ca²^+^ ions in the soil solution, thereby reducing calcium uptake by plant roots despite its presence in the extract.

Findings showed that spider plant treated with KM in season 1 had a highly significant response to magnesium. The control had lower leaf magnesium, but applying 30 kg/345 m^2^ KM increased leaf magnesium. These results confirm previous findings by Kassem and Marzouk (2010), who found high magnesium content in date palm (*Phoenix dactylifera*) when 45 kg KM was applied. This increase is attributed to the presence of magnesium (2.70 g/kg) in KM, which is adequate to enhance leaf magnesium. In contrast, Mhlontlo et al. (2008) reported no significant difference in the magnesium content of *Amaranthus* leaves when 2.5 t/ha KM was applied. Additionally, findings further showed no significant difference in leaf magnesium for both seasons when GKE was applied to spider plant. These findings correspond with those by Makhaye et al. (2021), who found that magnesium content in spider plant leaves was significantly reduced by the application of different Kelpak^®^ biostimulant rates (1:100, 1:40, and 1:20). In the current study, the dual application of KM and GKE did not show any significant difference in leaf magnesium in both seasons. Alginates are derived from undissolved calcium, magnesium, sodium, and potassium salts found in seaweed cell walls (Alam et al., 2022). Thus, this non-reaction may be due to the presence of alginates found in GKE, which reduced the availability of magnesium for plant uptake. Such binding effects may partially explain the lack of a significant magnesium response observed in the present study.

Findings showed that applying KM in both seasons resulted in a highly significant difference in leaf potassium. The application of 30 kg/345 m^2^ KM in both seasons increased leaf potassium; interestingly, as the KM amount increased to 60 kg/345 m^2^, leaf potassium decreased. These findings align with those of Barbosa et al. (2017), who found a significant increase in leaf potassium content with increasing doses of KM at 67.5 t ha^-1^. This significant increase when 30 kg/345 m^2^ KM was applied could be attributed to the available potassium (8.80 g/kg) in KM. Contrarily, Tran et al. (2018) found no significant difference in leaf potassium content despite increasing KM application rates. This can be ascribed to the enclosed environment, which could limit soil microbial activities (nutrient solubilization, decomposition, and root microbial interactions) responsible for nutrient uptake from the soil to the leaves (Bhattacharyya and Furtak, 2023). This is because in a glasshouse environment, variables such as restricted oxygen or carbon dioxide exchange, altered moisture levels, and reduced organic inputs can constrain microbial activity, thereby decreasing nutrient availability and absorption (Dorais et al., 2016). These limitations collectively reduce nutrient mineralization and root–microbe interactions, which are essential for efficient nutrient transfer from soil to plant tissues.

Moreover, no significant difference in leaf potassium was observed for both seasons when GKE was applied. In a corresponding study, Valizadehkaji and Mohammaei (2025) reported no significant difference in leaf potassium of mandarin (*Citrus reticulata*) when treated with seaweed biostimulant. This non-significant difference in leaf potassium may result from the soil having sufficient potassium, rendering the supplementary potassium from the GKE inconsequential for the spider plant’s development. When plant potassium demand is already met, uptake is tightly regulated, and additional external potassium inputs do not necessarily translate into increased leaf accumulation. In contrast, Makhaye et al (2021) found that the application of seaweed biostimulant (Kelpak^®^) increased leaf potassium of okra. This is because plants respond differently to seaweed extracts given their unique physiological and biochemical characteristics. Furthermore, leaf potassium increased in season 1 when treated with 30 kg/345 m^2^ KM + 1 mL/L GKE. This could be attributed to the high potassium concentration (3464 mg/L) in GKE.

A statistically significant difference in leaf sodium was observed in both seasons when KM was applied. The results further showed that as KM levels increased, leaf sodium also increased in both seasons. These findings align with those of Mmadi et al. (2019), who found that applying a 7:3 KM ratio increased leaf sodium levels. Contrarily, Hepburn et al. (2018) found that the application of KM did not affect the sodium content of swiss chard (*Beta vulgaris* var. *Cicla*). Results showed that there was a significant difference in leaf sodium when spider plant was treated with GKE in season 2. Moreover, season 2 showed a significant increase in leaf sodium when treated with 1 mL/L GKE + 30 kg/345 m^2^ KM. These findings are comparable with those of Osman and Salem (2011), who reported that the application of 0.4% seaweed extract (*Ulva lactuca*) significantly increased sodium uptake in sunflower (*Helianthus annuus*) when compared with the control. This increase can be attributed to the high sodium concentration (1268 mg/L) in GKE. Thus, like other seaweed extracts, GKE absorbs nutrients from the ocean water, which is abundant in sodium chloride. Hence, the application of GKE resulted in a highly significant leaf sodium uptake.

There was a highly significant leaf iron when season 1 was treated with KM. This is evident in season 1, as the application of KM increases, the iron content decreases. This corresponds with findings by Luthuli et al. (2024), who reported a significant difference in leaf iron after applying KM to tomatoes. Conversely, Mhlontlo et al (2007) found no significant difference in iron content in *Amaranthus* leaves when KM levels were increased from 0.3 – 0.6 t ha^-1^. Moreover, results showed that spider plant treated with 2 mL/L GKE in season 1 and 3 mL/L GKE in season 2 showed a highly significant effect on leaf iron content. Season 2 showed an increase in leaf iron when treated with 30 kg/345 m^2^ KM + 4 mL/L GKE. These findings align with Al-Shatri et al. (2019) who reported a significant difference in strawberry cv. Albion leaves when 8 g/L seaweed extract (alga 600) was applied. The increase in leaf iron can be attributed to the available iron (0.35 mg/L) in GKE.

The results showed that leaf manganese content differed significantly when KM was applied. This was observed in Season 1, whereas KM levels increased, manganese levels decreased, but interestingly, as KM increased in season 2, manganese content also increased. Similarly, Mmadi et al. (2019) reported that leaves showed a higher manganese content when treated with 8:2 and 7:3 KM. This could be attributed to KM containing a variety of minerals (6% nitrogen, 0.4% phosphorus, and 0.5% potassium) and bioactive substances, such as microbial activity, humic substances, and plant root exudates, which may improve manganese uptake (Marschner et al., 2003; Alejandro et al., 2020). Additionally, the application of GKE had a significant difference in manganese content. Season 1 showed that manganese increased when spider plant was treated with sole application of 2 mL/L GKE. These findings support those of Makoro (2015), who reported a significant effect on leaf manganese when 1:100 v/v and 1:500 v/v seaweed extract were applied in a tunnel house experiment. This could be attributed to the available manganese content (0.15 mg/L), which increased the nutrient’s uptake by the leaves. However, Makoro (2015) also found no significant difference in leaf manganese when 1:100 v/v and 1:500 v/v seaweed extract (*Ecklonia maxima*) were applied in a field trial. Findings showed that leaf manganese differed significantly when treated with the combination of 30 kg/345 m^2^ KM + 4 mL/L GKE. The significant results could be associated with the available manganese (0.15 mg/L) in GKE.

Findings showed that applying KM statistically increased leaf zinc content. In addition, the results showed that leaf zinc increased in season 1 when spider plant was treated with 60 kg/345 m^2^ KM. The increase in zinc content in leaves after KM application can be attributed to the available zinc (93.29 mg/kg) in KM, which increased soil zinc and plant uptake. In addition to direct zinc input, it is hypothesized that kraal manure improves zinc bioavailability by increasing organic matter inputs, which enhance chelation and reduce zinc adsorption to soil particles. The decomposition of organic residues and stimulation of soil microbial activity may promote zinc solubilization and facilitate root uptake. Similar mechanisms have been reported, in which organic amendments increased micronutrient availability by altering soil pH, cation exchange capacity, and root–microbe interactions. Meanwhile, in season 2, as 60 kg/345 m^2^ KM was applied, zinc remained the same. These results correspond with Luthuli et al. (2024), who reported a significant difference in leaf zinc content when tomatoes were treated with KM. Contrarily, Mmadi et al. (2019) found no significant difference in zinc content when potato (*Solanum tuberosum* L.) was treated with 7:3 and 8:2 KM ratios. This is because plants respond differently to KM treatments depending on soil type and pH. Additionally, the application of 4 mL/L GKE in season 2 showed an increase in the zinc content in spider plant leaves. Similarly, Dobromilska et al. (2008) reported an increase in zinc in tomato leaves when 1 mL/L seaweed extract (bio-algeen) was applied. This increase in leaf zinc uptake can be attributed to the available zinc amount (0.96 mg/L) in GKE. Contrarily, Al-Shatri et al. (2019) reported no significant difference in strawberry leaves when treated with different doses (0, 2, 4, and 8 g/L) of seaweed extract (Alga 600).

The significant interaction effects observed between GKE and KM for selected nutrients suggest a synergistic relationship between organic nutrient supply and biostimulant-induced physiological responses. Organic manures improve soil nutrient availability and microbial activity, while seaweed extracts enhance nutrient uptake efficiency by regulating phytohormonal signaling and root growth, and by modulating membrane transport processes (Eghball et al., 2002; Godlewska et al., 2016; Sosnowski et al., 2023). Such interactions are particularly important in nutrient-dense leafy vegetables, where balanced nutrient availability and uptake efficiency jointly determine leaf elemental composition.

Findings showed no significant differences in phosphorus when KM alone + GKE were applied. Likewise, Makoro (2015) found no significant difference in cowpea (*Vigna unguiculata* L. Walp) leaf phosphorus when 1:500 and 1:100 v/v seaweed extract (Technikelp) were applied. Conversely, Mhlontlo et al. (2007) found that applying more manure increased the absorption of nitrogen and phosphorus in vegetable leaves. Kraal manure comprises nitrogen, phosphorus, and potassium. Consequently, the greater the concentration of nitrogen (N), phosphorus (P), and potassium (K) in the manure, the more nutrients are available for plant absorption (Miles and Manson, 1998). Improved root biomass has been associated with enhanced nutrient uptake efficiency (Bell et al., 2024).

The CCA results revealed a strong correlation between plant growth parameters and leaf nutrients. Zinc consistently showed strong correlations with many plant growth parameters, including leaf yield, number of leaves, root biomass, shoot biomass, and chlorophyll content, indicating its essential role in plant development and its significant impact on leaf yield and number of leaves. Micronutrients zinc and iron are necessary for root growth (Nandal and Solanki, 2021). Meanwhile, chlorophyll content showed a positive correlation with potassium. Potassium contributes to nutritional balance, providing appropriate resources for chlorophyll production (Mostofa et al., 2022). Findings further showed that plant height and stem diameter were positively correlated with phosphorus, magnesium, nitrogen, and sodium. This suggests that nitrogen, phosphorus, sodium, and magnesium play a role in plant growth and development. The multivariate patterns revealed by CCA highlight the integrated nature of nutrient uptake and vegetative growth responses in spider plant. The close association between micronutrient accumulation and growth variables under combined GKE and KM treatments suggests synergistic effects between organic nutrient supply and biostimulant-induced physiological regulation, as previously reported in other crops (Godlewska et al., 2016; Sosnowski et al., 2023). Multivariate analysis therefore provided additional insight beyond univariate comparisons by illustrating treatment-driven nutrient–growth linkages.

Generally, findings showed that applying GKE in season 1 increased leaf yield and the number of leaves, which corresponds to the increased leaf zinc. Micronutrient-driven improvements in photosynthetic capacity were further supported by observed correlations among zinc, chlorophyll content, and potassium. The chlorophyll concentration improved with GKE treatments, which were provided by elevated zinc levels, thereby enhancing photosynthesis and promoting vegetative development, as evidenced by increases in leaf number and yield (Rehman et al., 2022). Potassium aids in preserving chloroplast functionality and regulating stomatal activity, hence facilitating photosynthesis (Imtiaz et al., 2023). Additionally, the application of GKE in season 1 increased chlorophyll content, which positively correlated with potassium. Adding GKE in season 1 increased stem diameter and plant height, which positively correlated with sodium, phosphorus, nitrogen, and magnesium. Furthermore, applying GKE in season 2 showed that root biomass, shoot biomass, leaf yield, and chlorophyll content increased with increasing leaf zinc. Giant kelp extract has been utilized to enhance the development and production of many crops. Moreover, more studies need to be conducted on how the combination of GKE with other easily accessible organic amendments affects commercial crop growth, yield and nutrients. Furthermore, application of KM in both seasons increased nitrogen, calcium, potassium, magnesium, iron, manganese, and zinc content in spider plant leaves, which led to the highly significant plant height, chlorophyll content, leaf yield, root biomass, shoot biomass and number of leaves. Applying KM improves crop growth and productivity by increasing nutrient availability and enhancing soil characteristics (Dordas, 2008; Matsi, 2011). However, fertilizer efficiency varies based on animal species, nutrition, digestibility, protein and fiber content, age, housing, environment, and production stage (Luthuli et al., 2024). In terms of nutrients, KM typically has the lowest nutrient density among other animal manures and exhibits a high moisture content (King, 2015). Therefore, this study used an eco-friendly seaweed extract which is a rich source of both micronutrients and macronutrients.

## 5 Conclusion

The study demonstrated that different levels of KM and GKE influenced the content of leaf micronutrients and macronutrients. The sole application of 2 mL/L GKE resulted in a significant increase in iron, manganese, and zinc in both seasons. Although GKE influenced some nutrients, it did not cause any significant changes in the concentrations of calcium, magnesium, potassium, or phosphorus in spider plant leaves. Furthermore, applying 30 kg/345 m^2^ KM increased the content of nitrogen, calcium, potassium, sodium, iron, zinc, and manganese in both seasons; however, it did not affect magnesium, iron, or phosphorus. Additionally, the combination of 2 mL/L GKE + 30 kg/345 m^2^ KM in both seasons increased leaf nitrogen, iron, manganese, and zinc. Kraal manure is a source of nutrients that is easily accessible to many farmers (particularly if sourced locally). Unlike chemical fertilizers, GKE is considered an environmentally friendly constituent. However, its availability is dependent on the ability to source and preparation of seaweed extract. The broader nutritional profile of KM may be beneficial for indigenous leafy vegetables such as spider plants. Likewise, GKE’s enhancement of iron, manganese, and zinc may be beneficial for micronutrient-deficient soils. Overall, the application of 30 kg/345 m² KM, either alone or in combination with GKE, emerged as the most effective strategy for improving nutrient composition and growth performance of spider plant under the conditions of this study. Lastly, further research is needed to examine the effects of GKE on the leaf elemental and phytochemical composition of various indigenous leafy vegetables, as well as to conduct controlled glasshouse experiments to validate and strengthen the observed positive responses to GKE.

## Data Availability

The raw data supporting the conclusions of this article will be made available by the authors, without undue reservation.
